# Positron Emission Tomography and Magnetic Resonance Imaging of the Brain in Fabry Disease: A Nationwide, Long-Time, Prospective Follow-Up

**DOI:** 10.1371/journal.pone.0143940

**Published:** 2015-12-02

**Authors:** Kirsten Korsholm, Ulla Feldt-Rasmussen, Henrik Granqvist, Liselotte Højgaard, Birgit Bollinger, Aase K. Rasmussen, Ian Law

**Affiliations:** 1 Department of Clinical Physiology, Nuclear Medicine and PET, Rigshospitalet, University of Copenhagen, Copenhagen, Denmark; 2 Department of Endocrinology, Rigshospitalet, University of Copenhagen, Copenhagen, Denmark; 3 Department of Radiology, Rigshospitalet, University of Copenhagen, Copenhagen, Denmark; Baylor Research Institute, UNITED STATES

## Abstract

**Background:**

Fabry disease is a rare metabolic glycosphingolipid storage disease caused by deficiency of the lysosomal enzyme α-galactosidase A—leading to cellular accumulation of globotriasylceramide in different organs, vessels, tissues, and nerves. The disease is associated with an increased risk of cerebrovascular disease at a young age in addition to heart and kidney failure.

**Objective:**

The objective of this study was to assess brain function and structure in the Danish cohort of patients with Fabry disease in a prospective way using 18-fluoro-deoxyglucose (F-18 FDG) positron emission tomography (PET) and magnetic resonance imaging (MRI).

**Patients:**

Forty patients with Fabry disease (14 males, 26 females, age at inclusion: 10–66 years, median: 39 years) underwent a brain F-18-FDG-PET-scan at inclusion, and 31 patients were followed with FDG-PET biannually for up to seven years. All patients (except one) had a brain MRI-scan at inclusion, and 34 patients were followed with MRI biannually for up to nine years.

**Image Analysis:**

The FDG-PET-images were inspected visually and analysed using a quantitative 3-dimensional stereotactic surface projection analysis (Neurostat). MRI images were also inspected visually and severity of white matter lesions (WMLs) was graded using a visual rating scale.

**Results:**

In 28 patients brain-FDG-PET was normal; in 23 of these 28 patients brain MRI was normal—of the remaining five patients in this group, four patients had WMLs and one patient never had an MRI-scan. In 10 patients hypometabolic areas were observed on brain-FDG-PET; all of these patients had cerebral infarcts/hemorrhages visualized on MRI corresponding to the main hypometabolic areas. In two patients brain-FDG-PET was ambiguous, while MRI was normal in one and abnormal in the other.

**Conclusion:**

Our data indicated that, in patients with Fabry disease, MRI is the preferable clinical modality—if applicable—when monitoring cerebral status, as no additional major brain-pathology was detected with FDG-PET.

## Introduction

Fabry disease is a rare X-linked recessive metabolic glycosphingolipid storage disease caused by a deficiency of the lysosomal enzyme α-galactosidase A (α-gal A) [1]. The glycosphingolipid substrate of α-gal A is globotriasylceramide (Gb3) which accumulates in e.g. endothelial cells, smooth muscles cells of the vascular system, renal epithelial cells, myocardial cells and central nervous system [2]. The most dominant clinical features of the patients include acroparaesthesia, angiokeratomas, corneal opacities and hypohidrosis, and with increasing age patients often develop cerebrovascular events (transient ischemic attacks and strokes) in addition to cardiovascular disease and renal failure.

A breakthrough in the treatment of patients with Fabry disease was the development of an effective and well-tolerated direct enzyme substitution in 2001[3,4], which shifted the management of the patients from a palliative to a causal active treatment.

Conventional magnetic resonance imaging (MRI) of the brain have shown that Fabry patients are at risk of severe and progressive white matter lesions (WMLs) at an early age in addition to cerebral infarcts and hemorrhages [5,6]—especially in the posterior cerebral circulation, probably due to dolichoectatic arteries [7,8]. However, the mechanism by which deficiency of α-gal A and glycosphingolipid-accumulation causes the Fabry vasculopathy is not completely understood [9]; a protrombotic state in Fabry disease has been confirmed [10], however, endothelial dysfunction and altered cerebral blood flow may also play a role [11]. In addition, emboli as a consequence of cardiac arrhythmia may also contribute.

The primary aim of our study was to assess long-term cerebral function in the nationwide Danish cohort of patients with Fabry disease using 18-fluoro-deoxyglucose (F-18 FDG) brain positron emission tomography (PET) scanning in addition to assessment of structural brain changes using MRI. The study was a descriptive, observational and prospective study initiated in relation to commencement of the treatment with enzyme substitution.

## Materials and Methods

### Patients

We studied 40 patients with Fabry disease (14 males, 26 females, age: 10–66 years, median 35 years (men) and 43 years (women), at inclusion). The study was approved by the National Ethics Committee (02-038/02, H-3-2014-FSP8), and informed consent was obtained from all participating subjects. The patients were followed at the Department of Endocrinology at Rigshospitalet, Copenhagen, Denmark, and the patients were examined with F-18 FDG-PET and MRI of the brain biannually for up to seven years (PET) / nine years (MRI) in addition to regular and systematic examinations for manifestations of Fabry disease as a part of the normal follow-up procedure at our hospital.

Fabry disease was confirmed in all patients by alpha-galactosidase A (*GLA*) mutation analysis, and the following mutations were seen: G85D (14 patients), A156T (11 patients), N34S (6 patients), G10694 (2 patients), I232T (2 patients), R342X (1 patient), D313Y (1 patient), G271S+D313Y (1 patient), c.369+3_c.547 (1 patient), G271S (1 patient) (see Table 1).

**Table 1:** Clinical and genetic features of the Fabry patients.

Patient no.	Age at inclusion	Sex	Mutation	Cerebro-vascular disease before inclusion	Cerebro-vascular disease during study	Cardio-vascular disease before inclusion	Cardio-vascular disease during study	Renal event before inclusion	Renal event during study	ERT
1	36	M	R342X	**-**	**-**	**-**	**-**	**-**	**-**	**+**
2	10	M	G85D	**-**	**-**	**-**	**-**	**-**	**-**	**+**
3	60	F	A156T	**+**	**-**	**U**	**+**	**-**	**-**	**+**
4	37	M	A156T	**+**	**-**	**-**	**+**	**D**	**KT**	**+**
5	63	F	A156T	**-**	**-**	**+**	**-**	**-**	**-**	**+**
6	58	F	A156T	**+**	**-**	**+**	**+**	**-**	**-**	**+**
7	41	F	A156T	**-**	**-**	**-**	**+**	**-**	**-**	**+**
8	38	F	G85D	**-**	**-**	**-**	**-**	**-**	**-**	**+**
9	42	F	A156T	**-**	**+**	**-**	**-**	**-**	**-**	**+**
10	37	M	A156T	**-**	**-**	**-**	**-**	**-**	**-**	**+**
11	15	M	N34S	**-**	**-**	**-**	**-**	**-**	**-**	**+**
12	15	F	A156T	**-**	**-**	**-**	**-**	**-**	**-**	**+**
13	33	F	A156T	**-**	**-**	**+**	**-**	**-**	**-**	**-**
14	27	F	G85D	**+**	**-**	**-**	**-**	**-**	**-**	**+**
15	34	F	N34S	**+**	**-**	**-**	**-**	**-**	**-**	**+**
16	54	F	A156T	**-**	**-**	**+**	**-**	**-**	**-**	**+**
17	34	M	G85D	**-**	**-**	**-**	**-**	**-**	**-**	**+**
18	18	M	N34S	**-**	**-**	**-**	**-**	**-**	**-**	**+**
19	59	F	G85D	**+**	**-**	**-**	**+**	**-**	**-**	**+**
20	48	F	N34S	**-**	**-**	**+**	**-**	**-**	**-**	**+**
21	55	F	G85D	**-**	**-**	**-**	**+**	**-**	**-**	**+**
22	35	M	G85D	**-**	**-**	**-**	**-**	**-**	**D, KT**	**+**
23	24	M	N34S	**-**	**-**	**-**	**-**	**-**	**-**	**+**
24	58	F	G85D	**+**	**-**	**+**	**-**	**-**	**-**	**+**
25	58	F	G85D	**-**	**+**	**-**	**-**	**-**	**-**	**+**
26	26	M	G85D	**-**	**-**	**-**	**-**	**-**	**-**	**+**
27	39	F	G85D	**-**	**-**	**-**	**-**	**-**	**-**	**-**
28	33	F	G85D	**-**	**-**	**-**	**-**	**-**	**-**	**+**
29	18	F	c.369+3_c.547	**-**	**-**	**-**	**-**	**-**	**-**	**+**
30	50	F	G10694	**-**	**-**	**-**	**-**	**-**	**-**	**+**
31	47	M	G10694	**-**	**-**	**-**	**-**	**-**	**-**	**+**
32	40	F	G85D	**-**	**-**	**-**	**-**	**-**	**-**	**+**
33	66	F	G271S	**+**	**-**	**+**	**-**	**-**	**-**	**+**
34	29	M	G85D	**-**	**-**	**-**	**-**	**D, KT**	**-**	**-**
35	35	M	N34S	**-**	**-**	**-**	**-**	**-**	**-**	**-**
36	44	F	D313Y	**-**	**-**	**-**	**-**	**-**	**-**	**-**
37	39	F	G271S + D313Y	**-**	**-**	**-**	**-**	**-**	**-**	**-** *
38	40	F	A156T	**-**	**-**	**-**	**-**	**-**	**-**	**-**
39	64	F	I232T	**-**	**-**	**-**	**-**	**-**	**-**	**-**
40	36	M	I232T	**-**	**-**	**-**	**-**	**-**	**-**	**+**

“+” yes

“-”no

*Chaparone-therapy.

ERT: Enzyme replacement treatment

Cerebrovascular disease: Infarct or hemorrhage

Cardiovascular disease: Arrhythmia, congestive heart failure or myocardial infarction

Renal event: Dialysis (D) or kidney transplantation (KT)

Unknown: U

Thirty-two patients were treated with enzyme replacement treatment (ERT). Initially all treated patients received agalsidase beta (Fabrazyme^®^, Genzyme (Cambridge, MA 02142)) 1 mg/kg intravenously every second week during the follow-up period. Because of a shortage of Fabrazyme^®^ in 2010/2011, most patients subsequently received agalsidase alfa (Replagal^®^, Shire (Hampshire, UK)) 0.2 mg/kg for the remaining follow-up period. One patient was treated with pharmacological chaperone therapy in a randomized trial. Seven patients did not receive any ERT (see Table 1).

Part of the patient cohort has been described previously [12–14].

### FDG-PET-Brain-Imaging

The distribution of the relative regional cerebral glucose metabolic rate (rCMRglc) was measured using F-18 FDG-PET-scanning. PET scans were performed with an eighteen-ring GE-Advance scanner (General Electric Medical Systems, Milwaukee, WI, USA) operating in 3D-acquisition mode, producing 35 image slices with an interslice distance of 4.25 mm. The total axial field of view was 15.2 cm with an approximate in-plane resolution of 5 mm [15]. Each patient received an intravenous bolus injection of approximately 200 MBq F-18 FDG while resting in the supine position with eyes covered and noise level kept at a minimum. After 30 min the patient was placed in the scanner, and the head fixed to restrict movements. A 10 min transmission scan was performed for attenuation correction followed by a 10 min 3-D emission scan. PET images of the FDG distribution were reconstructed using a 4.0 mm Hanning filter transaxially and an 8.5 mm Ramp filter axially. The same scanner was used in all patients for the sake of reproducibility, although the department acquired newer and better scanners during the study period.

### Magnetic Resonance Imaging

All patients (except one) underwent MRI of the brain around the time of the PET scans. Before 2008 all MRI-scans were performed on a 1.5 T Siemens Vision scanner, however, in 2008 the scanner was replaced by a 1.5 T Siemens Avanto scanner.

Sagittal T1-weighted images (TR 630 msec, TE 14 msec, flip-angle 90°, slice thickness 5 mm), axial double spin echo (TR 3703 msec, TE 22/90 msec, flip-angle 180°, slice thickness 5 mm) and coronal FLAIR-images (TR 9000 msec, TE 105 ms, flipangle 180°, slice thickness 3 mm) were obtained before contrast-injection, and sagittal and axial T1-weighted images (TR 588 msec, TE 17 ms, flip-angle 90°, slice thickness 5 mm) were obtained after contrast-injection (Multihance 0.2 ml/kg iv). Contrast was not given to patients with impaired kidney function.

### Data Analysis

The FDG PET images were registered to the patients T1- and T2-weighted MRI scans, oriented parallel to the cranial base, and evaluated visually in coronal, sagittal and transaxial sections using the “3D” program in the Siemens Multimodality Workplace by a specially trained nuclear medicine physician (IL). In addition to visual analysis, a quantitative 3-dimensional stereotactic surface projection analysis (Neurostat) was employed that allows direct visualization of the extent and topography of FDG uptake abnormalities [16]. This procedure involved reconstruction of the images with an 8 mm Hann filter, warping them to a standard stereotactic space and projecting regional cortical activity normalized to the cerebellum unto the cerebral surfaces. The surface projections were subsequently compared voxel-by-voxel to three cohorts of age-matched groups of healthy subjects depending on the patients’ age (19–34 years, 30–60 years, and 55–90 years). Significant regional deviation from the mean was expressed by a Z-score using a threshold value of Z >3.09 (p < 0.001, one sided).

Progression of any pathology on the PET-scans were noted and described.

A trained neuroradiologist (BB) evaluated all MRI scans. WMLs were defined as bright lesions of ≥ 5 mm on FLAIR images, and WML severity was graded on a 4-rate scale [17,18]. Images with no or a single punctate lesion were classified as 0, multiple punctate lesions were classified as 1, beginning confluence of lesions was classified as 2 and large confluent lesions were classified as 3.

Lesion progression was noted and classified as *minor* if lesion-load had increased by one to four additional punctuate lesions on follow-up, and lesion progression was noted as *marked* if ≥ five punctuate lesions had developed or if there was a transition to confluent lesions [19,20]. In addition, infarcts, hemorrhages and other vascular pathology were described.

## Results

Forty patients underwent a brain FDG-PET-scan at inclusion, and 31 patients were followed with brain FDG-PET ranging from two to four scans per patient for up to seven years (Table 2). Nine patients were only scanned once.

**Table 2:** FDG-PET and MRI-features of the Fabry patients.

Patient no.	No of brain PET/brain MRI	Brain PET pathology	WML on any brain MRI	Other brain MRI pathology incl. infarcts	Progression of pathology on PET/MRI ^c^
1	3/5	-	-	Minimal hyperintensity of both pulvinar on T1	- / -
2	4/4	-	-	-	- / -
3	4/4	Hypometabolic areas corresponding to infarcts; thalamocortical diaschisis+crossed cerebello-cortical disachisis	WML grade 2	Multiple infarcts in right frontal region, left basal ganglia and left cerebellar hemisphere	Aggravation of crossed cerebello-cortical diaschisis / -
4	4/4	Hypometabolic areas corresponding to infarcts and in left mesial temporal region.	WML grade 2	Multiple infarcts in both frontal regions and cerebellum	Development of reduced metabolism in left mesial temporal region / -
5	4/2	Probably normal ^a^	WML grade 2	-	- / -
6	4/3	Discretely reduced metabolism in frontal areas bilaterally	WML grade 2	Infarct in right frontal region	- / -
7	4/3	-	WML grade 1	-	- / -
8	2/3	-	WML grade 1	-	- / Minor progression (WML grade 0 to grade 1)
9	3/4	Hypometabolic area in right corona radiate corresponding to infarct	WML grade 1	Infarct in right corona radiate	Development of a hypometabolic area corresponding to infarct / Infarct in corona radiata. Marked progression (5 new focal WMLs)
10	4/4	-	-	-	- / -
11	4/5	-	-	-	- / -
12	4/5	-	-	-	- / -
13	3/3	-	-	-	- / -
14	4/5	Hypometabolic area in pons (infact), diffuse hypometabolism in both cerebellar hemispheres	-	Infarct in left side of pons	- / -
15	3/2	Hypometabolic area in right insula (infact) including adjoining temporal cortex	-	Infarct in right insula with gliosis. Occluded right carotid artery	- / -
16	3/3	-	-	-	- / -
17	3/2	-	-	Small arachnoidal cyst	- / -
18	3/3	-	-	-	- / -
19	3/3	Hypometabolic areas corresponding to infarcts in frontal cortex and thalamus.	WML grade 1	Multiple infarcts in frontal regions and left thalamus	- / Minor progression in WML (one new focal WML)
20	3/3	-	-	-	- / -
21	3/3	-	WML grade 2	-	- / -
22	1/2	Probably normal ^b^	-	-	n.a. / -
23	3/3	-	-	-	- / -
24	2/2	Hypometabolic area in left thalamus corresponding to infarct	WML grade 3	Infarct in left thalamus	- / -
25	2/2	Hypometabolic area in cerebellum corresponding to tissue loss. Cerebello-cortical diaschisis	WML grade 1	Hemorrhage in left cerebellar hemisphere	Development of hypometabolic area corresponding to tissue loss. Development of cerebello-cortical diaschisis / Cerebellar hemorrhage
26	2/2	-	-	-	- / -
27	2/2	-	-	-	- / -
28	2/2	-	-	-	- / -
29	2/2	-	-	-	- / -
30	1/1	-	-	-	n.a. / n.a.
31	1/2	-	WML grade 2	-	n.a. / -
32	2/2	-	-	-	- / -
33	2/2	Hypometabolic areas corresponding to infarcts. Reduced metabolism in right temporooccipital area	WML grade 2	Infarcts in brainstem and right basal ganglia	- / -
34	1/0	-	n.a	n.a	n.a. / n.a.
35	1/2	-	-	-	n.a. / -
36	1/1	-	-	-	n.a. / n.a.
37	1/1	-	-	-	n.a. / n.a.
38	2/2	-	-	-	- / -
39	1/1	-	-	-	n.a. / n.a.
40	1/1	-	-	-	n.a. / n.a.

***n*.*a*.** *not applicable*

***-*** *WML grade 0 or pathology not present*

***- / -*** *no changes in neither PET nor MRI*

^**a**^ Patient no. 5: symmetrical mildly reduced activity parietotemporally bilaterally

^**b**^ Patient no. 22: symmetrical mildly reduced activity in both thalami *Cont*.

^**c**^ Progression of pathology on either PET or MRI was detected in the following patients:

Patient no. 3: PET-study period: seven years

Patient no. 4: PET-study period: six years.

Patient no. 8: MRI study period: three years

Patient no. 9: PET-study period: five years. MRI study period: seven years.

Patient no. 19: MRI study period: five years

Patient no. 25: PET/MRI study period: two years.

Patients underwent brain MRI biannually performed around the time of the PET-scan, and were followed for up to nine years ranging from two to five scans per patient (five patients only had one MRI-scan during the study period, and one patient never had an MRI-scan performed) (Table 2)

In 28 patients FDG-PET scan of the brain was normal both initially and during follow-up, that is, no areas of hypo- or hypermetabolism were detected compared to an age-matched control-group (Table 2). MRI of the brain was normal in 23 of these 28 patients (apart from minor findings in pts. no. 1 and 17 where hyperintensity of the pulvinar on T1-weighted images, respectively an arachnoidal cyst was detected). Of the remaining five patients in this group, one patient never had an MRI-scan, four patients had WMLs (grade 1 or 2), and progression of WMLs was seen in one of these patients (pt. no. 8) (Table 2).

In two patients (no. 5 and 22) FDG-PET of the brain was probably normal (one patient showed symmetrically mildly reduced activity parieto-temporally bilaterally which remained stable over seven years (four PET-scans)), and one patient showed symmetrically mildly reduced activity in both thalami (only PET-scanned once). The patient with mildly reduced activity parieto-temporally bilaterally had WMLs grade 2 on MRI which did not progress during follow-up (short follow-up: 2 years). The patient with mildly reduced activity in both thalami had two normal MRI of the brain.

In 10 patients hypometabolic areas were observed on FDG-PET of the brain either at inclusion or at follow-up. All of these patients had cerebral infarcts/hemorrhages visualized on MRI corresponding to the main hypometabolic areas. In six of these patients the metabolic changes were stationary over time. One of the patients with stationary metabolic defects (patient no. 19) showed progression on MRI (minor progression in WML, one new focal WML). The remaining patients in this group developed no new structural changes detectable with MRI.

**Fig 1:**
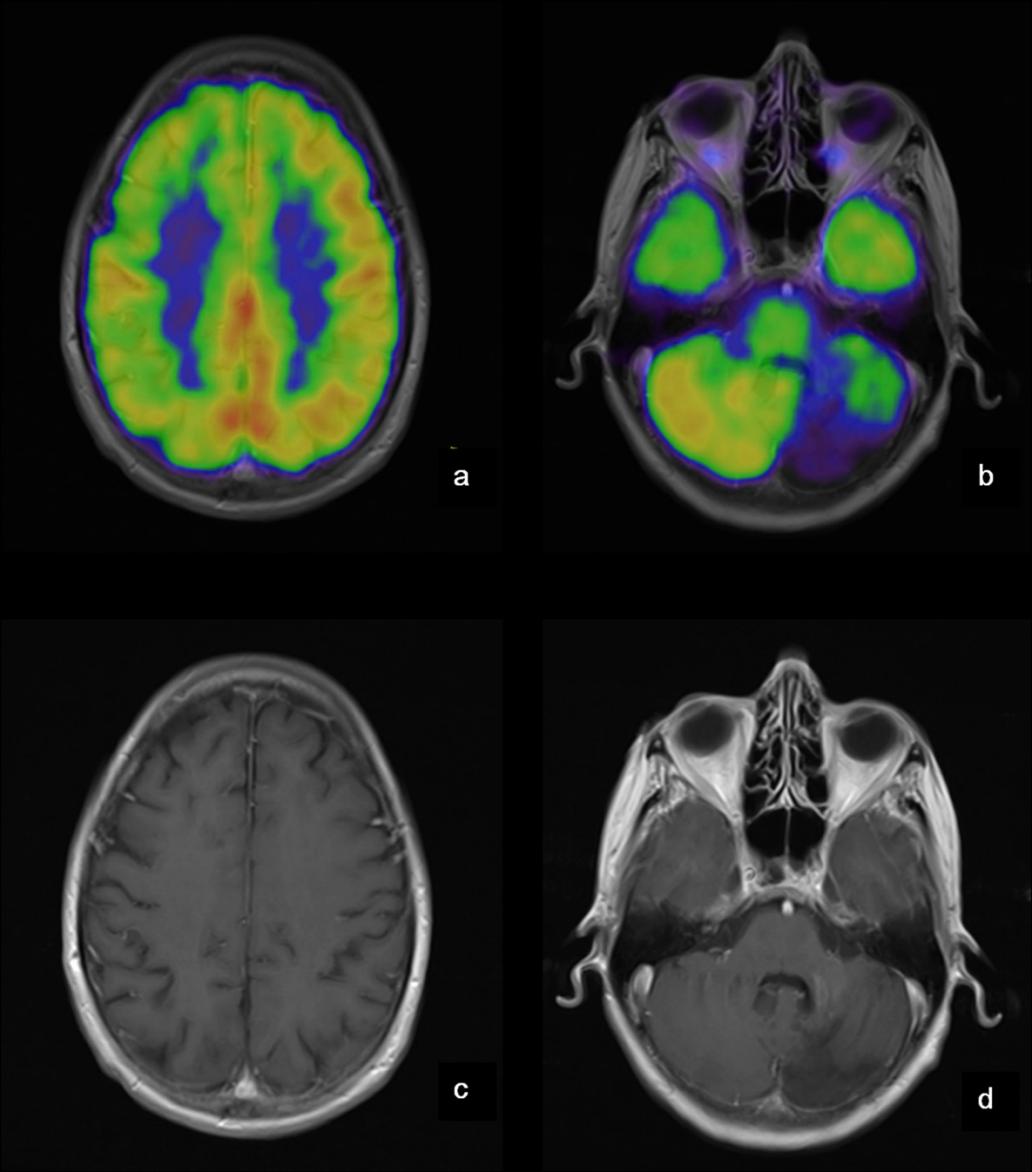
PET and MRI of the brain of patient no 25. Patient no. 25 suffered from a cerebellar hemorrhage and developed a hypometabolic area corresponding to tissue loss in the left cerebellar hemisphere (b + d) in addition to a cerebello-cortical diaschisis (a + b). **a**: Cortex (MRI fusioned with PET)–decreased activity in the right hemisphere. **b**: Cerebellum (MRI fusioned with PET)–decreased activity in the left cerebellar hemisphere. **c**: Cortex (MRI)–no structural changes. **d**: Cerebellum (MRI)–sequelae after hemorrhage.

**Fig 2:**
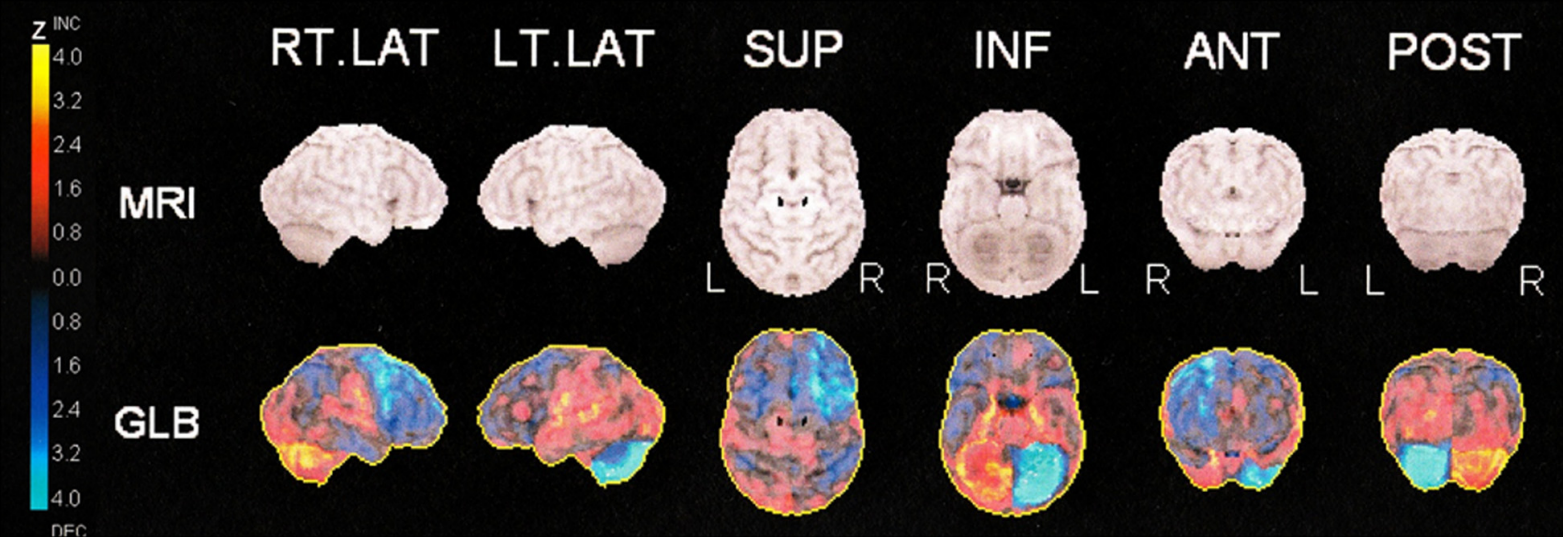
Quantitative 3-dimensional surface projection-analysis of FDG-uptake in patient no 25. PET-images of patient no. 25 are normalized to whole brain using a database of normal subjects and scaled to Z values from - 4.0 to 4.0 (Neurostat). Projections of Z values are shown onto, respectively, the right and left lateral hemispheric surfaces, the superior and inferior surfaces, and the anterior and posterior surfaces. The uptake in the left cerebellar hemisphere is reduced in addition to uptake in the right frontal cortex. MRI surface projections are presented for anatomical reference in a standard stereotactic space

In four patients progression of pathology of glucose metabolism was seen on FDG-PET during follow-up: One patient (no. 25) suffered from a cerebellar hemorrhage and developed a hypometabolic area corresponding to tissue loss in the cerebellum (visualized by MRI) in addition to a cerebello-cortical diaschisis (see Figs 1 and 2).

Another patient (no. 9) suffered from an infarct in the right corona radiata with tissue loss seen on MRI and corresponding hypometabolism on FDG-PET. In addition, marked progression with five new focal WMLs was seen in this patient. Both of these patients had a normal FDG-PET scan initially.

A third patient (no. 3) developed a more pronounced crossed cerebellar diaschisis without any new structural changes on MRI, and the fourth patient (no. 4) developed slightly reduced glucose metabolism in the left temporal region mesially also without any evidence of new structural changes on MRI.

In addition to the hypometabolic areas corresponding to infarcts, minor pathology was seen on FDG-PET in a few patients e.g. diffuse hypometabolism in both cerebellar hemispheres (patient no. 14 (infarct in pons)) and a hypometabolic area in adjoining temporal cortex (patient no.15 (infarct in insula)) (see Table 2).

In total, WMLs grade 1–3 were seen in 13 of 40 patients (32.5%) (median age 58 years at inclusion, range 37–66). Lesions were classified as grade 1 in five patients, as grade 2 in seven patients, and as grade 3 in one patient (Table 2). Progression of WMLs was seen in three patients (follow-up: range 3–7 years), and one of these patients (no. 9) also developed a new infarct.

Cerebral infarcts were detected on MRI in nine of 40 patients (22.5%), and cerebral hemorrhage in one patient (2.5%).

Eight of the 10 patients with infarcts/hemorrhages had WMLs.

Abnormal bright signal in the pulvinar on T1-weighted images was seen in one patient (patient no 1, male).

Nine of the female patients (34.6%) had cerebrovascular disease, and one (7.1%) of the male patients (Table 1).

Seven patients had a history of cardiovascular disease (arrhythmia, congestive heart failure or myocardial infarction) prior to inclusion, and six patients developed cardiovascular disease during the study. In the group of patients with cerebral infarcts/hemorrhages (10 patients), six patients also had cardiovascular disease.

Two patients had renal disease prior to inclusion (one patient was in renal dialysis; one patient had undergone kidney transplantation). The patient in renal dialysis was transplanted during the study. A third patient begun renal dialysis during the study and was later transplanted (see Table 1). Only one of the patients with infarcts also had renal disease.

## Discussion

Cerebrovascular events are common in patients with Fabry disease and in order to monitor long-time brain function and structure, our cohort of patients with Fabry disease underwent FDG-PET- and MRI-scans of the brain biannually in a prospective follow-up study. Overall, we found that both modalities detect infarcts, however, more detailed information about brain structure and pathology are discovered with MRI including WMLs.

Cerebral glucose metabolism in patients with Fabry disease has previously been investigated by Moore et al [21] who found that the *mean* global cerebral glucose metabolism (CMRGlu) was not significantly different between patients with Fabry disease and controls. This was in accordance with our results, where we find a normal cerebral glucose metabolism distribution in the group of patients without infarcts/hemorrhages.

However, Moore et al [21] also reported that glucose metabolism was decreased in some regions of white matter including areas with WMLs seen on the FLAIR-sequence on MRI. In our study WMLs were detected in 13 patients, however, we did not see any clear or obvious changes in glucose-metabolism corresponding to the WMLs. This could in part be explained by a small size of the WMLs and resolution limitations. In the analysis of glucose metabolism we used Neurostat as it has been shown that aid from this program increases sensitivity and reduces intra-observer variability in the identification of hypo- or hypermetabolic areas [22].

In another study, also by Moore et al [23], it was demonstrated that resting *global* cerebral blood flow (CBF) was not different between patients and controls; however, in specific brain regions (the brain stem, cerebellum, bilateral temporal, posterior occipital and inferior frontal regions) an increased regional CBF was seen in patients. After ERT the resting global CBF fell significantly in the treated group, whereas in the placebo group it increased together with an increase in regional CBF. Therefore, it was speculated if the elevated CBF in the placebo group could contribute to endothelial dysfunction and vessel wall dilation and result in an abnormal flow state together with an increased risk of emboli and thrombosis.

As Fellgiebel states [5] it is unknown if ERT has long term protective effects against cerebrovascular events and only longitudinal studies including patients under ERT as well as untreated patients can answer this question. Unfortunately in our study, only six patients were FDG-PET-scanned both before and after commencement of treatment with ERT, rendering a comparison of scans before and after treatment with ERT impossible; in addition development of pathology detected with PET was only seen in four patients (follow-up 2–7 years); with MRI development of pathology was also detected in four patients, however not the same (follow-up 2–7 years).

A third study—also by Moore et al [24] did not report any abnormalities in visual regional CBF reactivity suggesting normal neurovascular coupling mechanisms in patients with Fabry. However, a prolonged cerebral vascular response time after acetazolamid challenge was seen in patients with Fabry, suggesting an abnormality on the luminal side of the vessels. As we did not investigate cerebral blood flow in our group of patients, we cannot collate the results.

In our cohort of patients cerebral infarcts were detected on MRI in nine of 40 patients (22.5%), cerebral hemorrhage in one patient (2.5%), and WMLs were seen in 13 of 40 patients (32.5%).

Median age of patients with WMLs was 58 years in comparison to 39 years (whole group). Lesions were punctate (grade 1) in five patients (12.5%) and confluent (grade 2 or 3) in eight patients (20.0%).

In other studies of patients with Fabry disease focal WMLs were detected in respectively 22% and 28% of patients, and confluent WMLs were detected in respectively 33% and 17% of the patients [25,26]. These numbers corresponded fairly well to our result. Twelve patients in the latter study had two brain MRIs during the study period, and in eight patients no changes occurred during a follow-up of approximately 2 years, whereas new WMLs were identified in four patients [26]. This was in reasonable accordance with our results, where new lesions were identified in 3 of 40 patients during follow-up (range 3–7 years).

Crutchfield [8] et al found that lesion burden detectable by MRI increased with age, and in addition they found that 37.5% of patients with WMLs had had a symptomatic stroke and 21.9% had had a transient ischemic attack (TIA), whereas only 6.2% of patients without WMLs on MRI had had a TIA and none had had a symptomatic stroke. In our cohort 13 patients had WMLs and 8 of these (69%) had suffered from a cerebrovascular event. Twenty-seven patients had no WMLs and two of these (7%) had suffered from a cerebrovascular event.

Fellgiebel et al [5] reported that seven of 25 patients (28%) with Fabry had markedly elevated WML-volumes, however, mean volume of WMLs between the rest of the patients was not significantly different from the control group. In addition they also studied mean diffusivity with MRI and mean diameter of the basilar artery, and found that basilar artery diameter was significantly larger in patients with Fabry than in controls. Global mean diffusivity was significantly elevated in Fabry patients compared to controls, and this has also previously been reported even in patients without WMLs, suggesting a sensitive marker of early brain involvement [27].

The pulvinar sign (a bilateral neuroradiological sign detected in T1-weighted MRI of the lateral part of the pulvinar—mostly seen in male patients—and considered expression of hyperperfusion with subsequent dystrophic calcification of the pulvinar [28]) was seen in one of our patients (patient no 9). In a study by Burlina et al [29] the pulvinar sign was found in five male patients (out of 36 patients), and the sign was associated with cardiac disease and severe kidney involvement; however, in our study the patient with the pulvinar sign had no cardiovascular or renal disease. Reisin et al. [26] reported that the pulvinar sign was seen in one patient among 36 adult patients, which is in accordance with our results.

Nine of the patients with cerebrovascular disease were female (of a total of 26 female patients), one was male (of a total of 14 male patients) (Table 1). In the Fabry Outcome Survey (FOS) the prevalence of stroke or transitory cerebral ischemia was also higher in female (15.7%) than in male (11.1%) patients [30].

In the group of patients with pathology detected with MRI, however with normal or probably normal FDG-PET, one patient—with progression of WMLs during the study period—suffered from a cerebral infarct years later—this corresponds well to the fact that there is a known association between WMLs and stroke [31]. Another patient with WMLs and normal FDG-PET died a year after the study period—however, the cause of death was unknown.

In conclusion, we found that the majority of hypometabolic areas on FDG-PET corresponded to cerebral infarcts or hemorrhages detectable on conventional MRI of the brain. No areas with hypermetabolism on FDG-PET were detected. There were few and minor findings on FDG-PET not detectable on MRI (diaschisis, discrete hypometabolism), however, FDG-PET provided no additional clinical relevant information. As MRI can diagnose brain pathology and is without radiation in contrast to FDG-PET, where the patient is given a considerable dose of radiation, we suggest MRI as the preferable clinical modality when monitoring cerebral status in patients with Fabry disease.

However, as more and more patients with Fabry disease have implantable cardioverter defibrillators or pacemakers (ICDs) due to heart disease, making MRI difficult to apply, FDG-PET/CT might be used in these patients, when monitoring brain function.

To our knowledge a prospective brain FDG-PET-study spanning over several years has not yet been published, and we find it of great importance to point out that FDG-PET is not the clinical modality to choose when monitoring brain status in patients with Fabry disease unless MRI of the brain is not applicable.

## Ethical Standards

The study was approved by the Regional Ethics Committee (02-038/02, H-3-2014-FSP8), and informed consent was obtained from all participating subjects.
